# Novel tongue-positioning device to reduce tongue motions during radiation therapy for head and neck cancer: Geometric and dosimetric evaluation

**DOI:** 10.1371/journal.pone.0291712

**Published:** 2023-09-21

**Authors:** Seongmoon Jung, Bitbyeol Kim, Sung Young Lee, Won Ick Chang, Jaeman Son, Jong Min Park, Chang Heon Choi, Joo Ho Lee, Hong-Gyun Wu, Jung-in Kim, Jin Ho Kim

**Affiliations:** 1 Department of Radiation Oncology, Seoul National University Hospital, Seoul, Republic of Korea; 2 Institute of Radiation Medicine, Seoul National University Medical Research Center, Seoul, Republic of Korea; 3 Biomedical Research Institute, Seoul National University Hospital, Seoul, Republic of Korea; 4 Department of Nuclear Engineering, Ulsan National Institute of Science and Technology, Ulsan, Republic of Korea; 5 Department of Radiation Oncology, Seoul National University College of Medicine, Seoul, Republic of Korea; Chung-Ang University Gwangmyeong Hospital, REPUBLIC OF KOREA

## Abstract

This study aimed to assess the performance of a tongue-positioning device in interfractional tongue position reproducibility by cone-beam computed tomography (CBCT). Fifty-two patients treated with radiation therapy (RT) while using a tongue positioning device were included in the study. All patients were treated with 28 or 30 fractions using the volumetric modulated arc therapy technique. CBCT images were acquired at the 1^st^, 7^th^, 11^th^, 15^th^, 19^th^, 23^th^, and 27^th^ fractions. Tongues on planning computed tomography (pCT) and CBCT images were contoured in the treatment planning system. Geometric differences in the tongue between pCT and CBCT were assessed by the Dice similarity coefficient (DSC) and averaged Hausdorff distance (AHD). Two-dimensional *in vivo* measurements using radiochromic films were performed in 13 patients once a week during sessions. The planned dose distributions were compared with the measured dose distributions using gamma analysis with criteria of 3%/3 mm. In all patients, the mean DSC at the 1^st^ fraction (pCT versus 1^st^ CBCT) was 0.80 while the mean DSC at the 27^th^ fraction (pCT versus 27^th^ CBCT) was 0.77 with statistical significance (*p*-value = 0.015). There was no statistically significant difference in DSC between the 1^st^ fraction and any other fraction, except for the 27^th^ fraction. There was statistically significant difference in AHD between the 1^st^ fraction and the 19^th^, 23^th^, and 27^th^ fractions (*p*-value < 0.05). *In vivo* measurements showed an average gamma passing rate of 90.54%. There was no significant difference between measurements at the 1^st^ week and those at other weeks. The tongue geometry during RT was compared between pCT and CBCT. In conclusion, the novel tongue-positioning device was found to minimize interfractional variations in position and shape of the tongue.

## Introduction

During radiation therapy (RT) in patients with head and neck cancer, several tongue-positioning devices have been clinically used. Tongue-positioning devices are used to reduce radiation dose delivered to the oral tongue and inter- and intra-fractional motion errors associated with RT for head and neck cancer. Using tongue-positioning devices during RT for head and neck cancer could dramatically decrease probability of oral complications of RT such as radiation-induced oral mucositis [1, 2]. In a recent study, in 2020, a significant improvement in taste impairment and difficulty in swallowing/chewing has been shown when using tongue-positioning device [3].

Various tongue positioning devices have been used in clinics. The dosimetric evaluations of conventional tongue bite or tongue depressor have been evaluated [4–8]. The conventional method includes tongue-positioning device made of paraffin wax as well as the tongue-positioning device made of silicone material using mold and casting method [9–11]. In 2022, it was reported that the use of positioning stent to reduce the irradiation dose to the palate resulted in a reduction of radiation-induced oral mucositis on the palate [10]. Several three-dimensional (3D) printed customized tongue devices were suggested to take the patient-specific structures into account, and dosimetric efficacies were also reported [6–9, 12–16]. In 2022, the interfractional head-up and–down motions in the thermoplastic mask with and without 3D printed tongue-positioning devices were investigated using magnetic resonance imaging [14]. It was revealed that head motions with the tongue-positioning devices decreased significantly compared to the motions without tongue-positioning devices [14]. In 2021, dosimetric characteristics and setup stability of a patient-specific semi-customized tongue-positioning device were compared with those of a standard mouthpiece. It was investigated that the dose of median mucosa of the tongue was significantly reduced when using the patient-specific semi-customized tongue-positioning device [15]. However, these 3D printed tongue-positioning devices require fabrication time and the procedures are labor-intensive. If a tongue-positioning device requires molding and casting, a considerable amount of time would be needed to fabricate the device.

Commercially available tongue-positioning devices used for RT also exist. In 2020, a prospective study was performed to assess non-inferiority of customized oral stents made using 3D printing compared to manually fabricated stents and a commercially available one (TrueGuard^TM^ manufactured by Bionix) [9, 17]. No significant difference was observed between 3D printed oral stent and TruGuard^TM^ in terms of inter-incisal opening and position reproducibility [9]. A dosimetric advantages of using a commercial device (GrayDuck Stent^TM^ manufactured by CIVCO) was also presented by showing several treatment plans [18]. Although the GrayDuck Stent^TM^ and TruGaurd^TM^ are convenient to use, they do not have enough room for patient-specific customization. The GrayDuck Stent^TM^ with two different types of paddles moves the tongue to the left, right, or bottom [19]. Although many researchers have compared treatment plans and clinical outcomes of tongue-positioning device, none have assessed the actual interfractional tongue positions during treatment course.

This study aimed to assess the geometrical changes in interfractional tongue positions when using a tongue-positioning device by comparing the tongue contours on cone-beam computed tomography (CBCT) and planning CT (pCT) images. A new commercial tongue-positioning device (BinkieRT^®^, Paprica Lab., Ltd.) has been recently released and used in this study [20, 21]. Furthermore, *in vivo* dosimetry was performed to verify that the delivered doses were reproducible during the RT course.

## Materials and methods

### Ethics statement

All procedures performed in study involving human participants were in accordance with the ethical standards of the institutional review board (IRB approval No. D-2008-040-1148) and with the 1964 Helsinki declaration and its later amendments or comparable ethical standards. Fifty-two patients who were diagnosed with malignant tumors in the head and neck region and underwent RT between May 2021 and November 2021 were recruited. We had access to information which could identify individual participants during data collection. Informed written consent was obtained from all individual patients included in this study.

### Patient treatment

**Fig 1:**
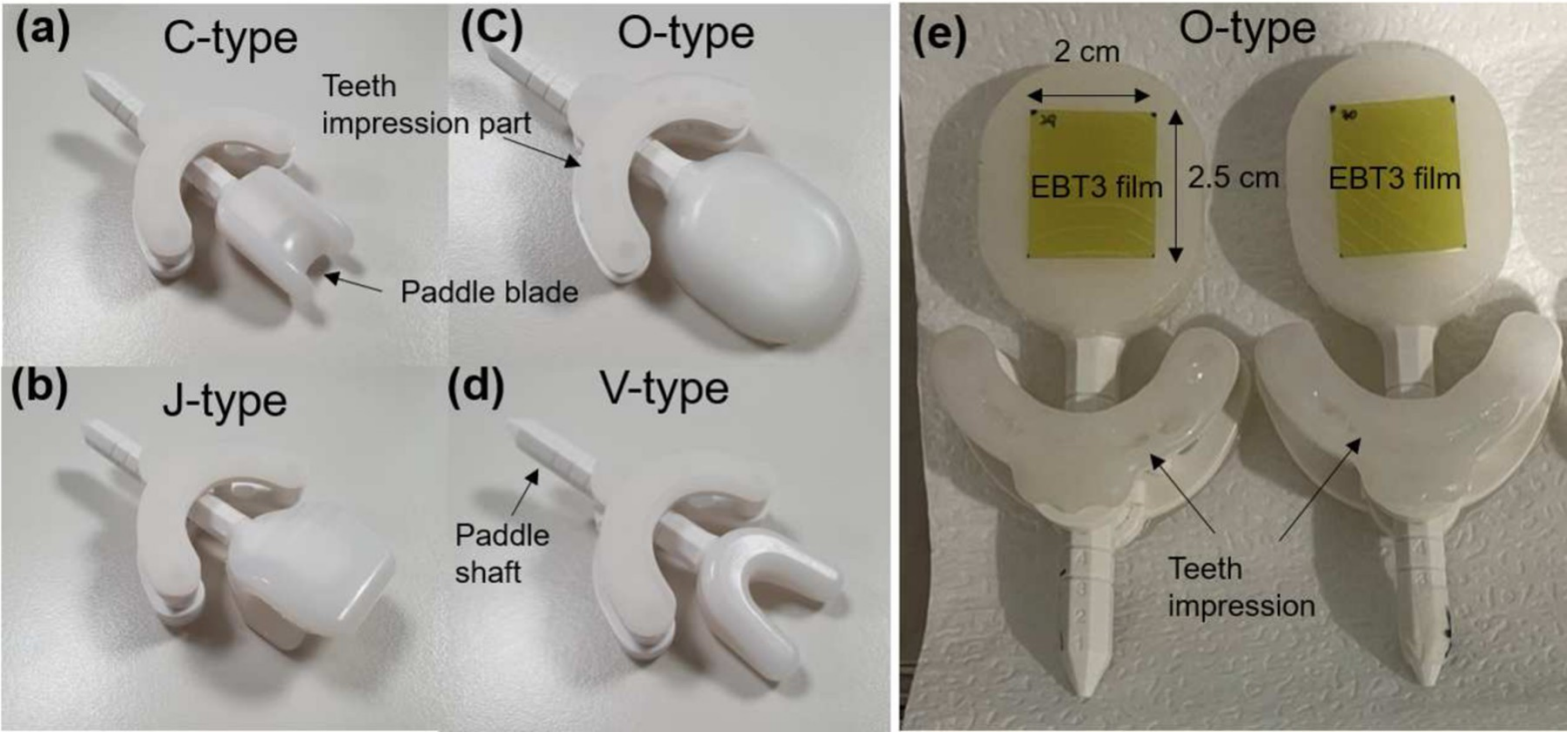
Photographs of tongue positioning devices with different types of paddle blade. (a) C-type, (b) J-type, (c) O-type, and (d) V-type. (e) O-type tongue positioning device attached to external beam therapy 3 (EBT3) film was used for *in vivo* measurements.

**Fig 2:**
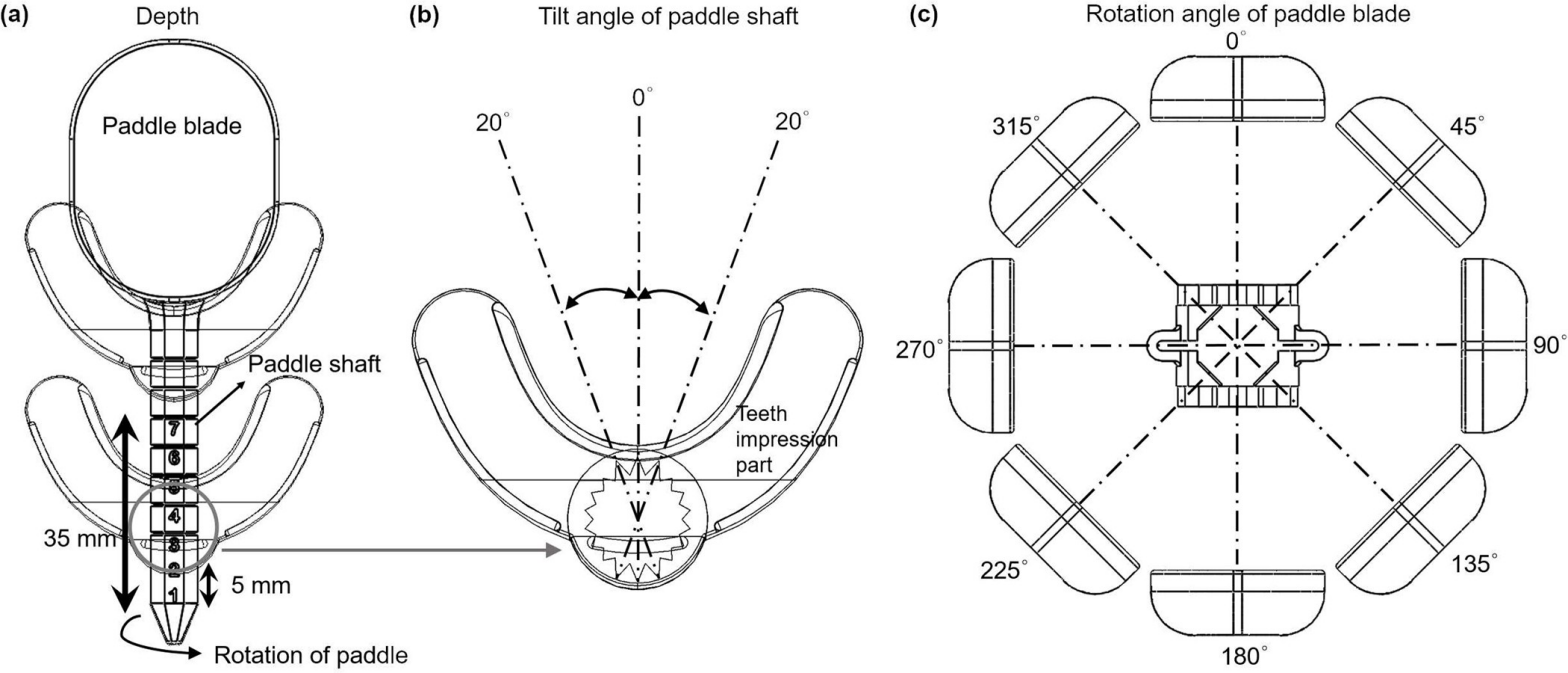
Illustrations of positioning devices showing various achievable positions. (a) Depth, (b) tilt angle of paddle shaft and (c) rotation angle of paddle blade.

**Fig 3:**
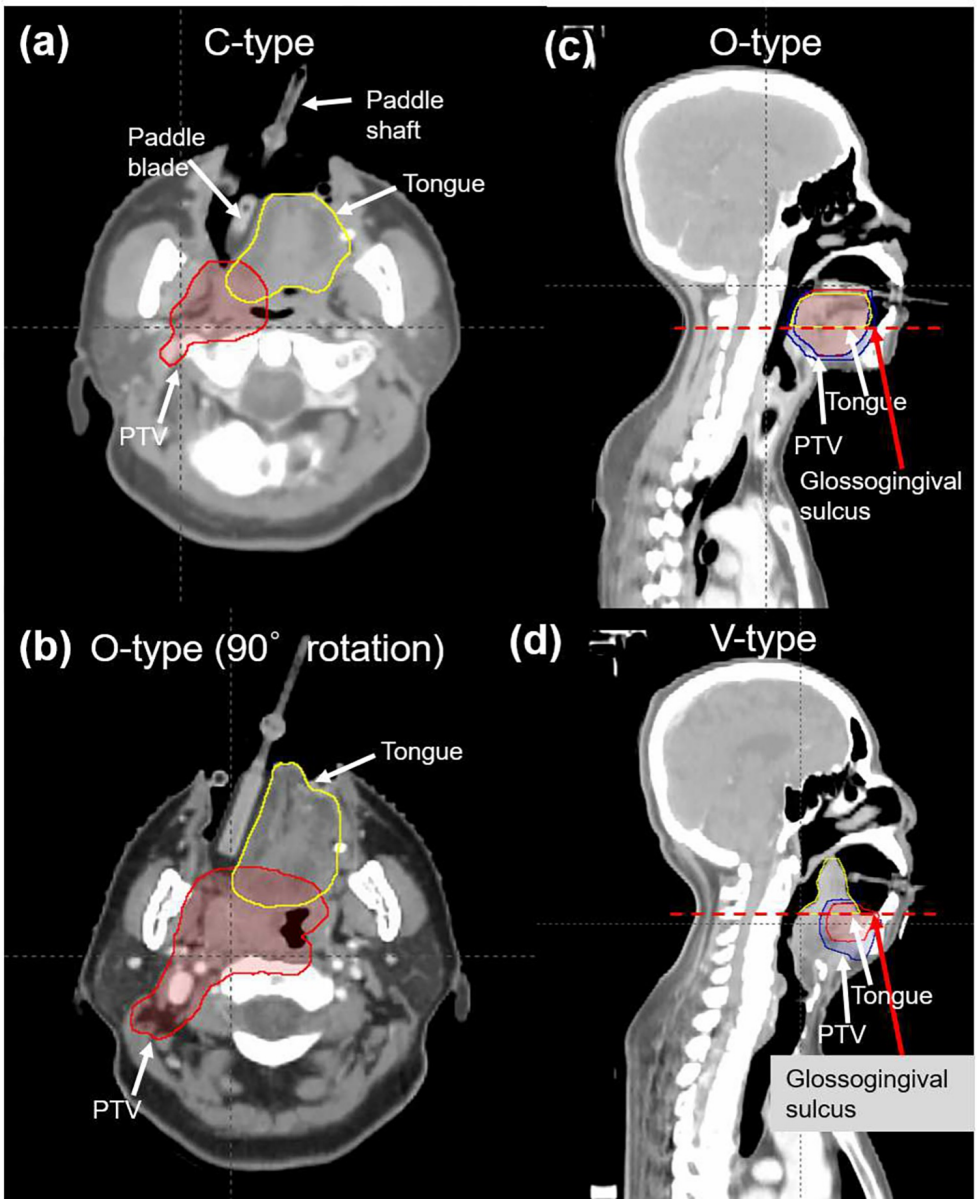
Planning CT images of patients with tongue-positioning devices. (a) axial view of C-type insertion, (b) axial view of O-type with 90° rotation insertion, (c) sagittal view of O-type insertion, and (d) sagittal view of V-type insertion. The red dotted horizontal line shows the bottom plane of the tongue.

The clinical target volumes (CTVs) included one or several parts of the tongue, floor of the mouth, oropharynx, larynx, nasal cavity, oral cavity, or maxillary sinus. All patients used the tongue-positioning device during computed tomography (CT) simulation and treatment. The patient characteristics and the type of tongue-positioning device used are listed in Table 1. Fig 1 shows a photograph of the tongue positioning device used in this study. The BinkieRT^®^ has four different types of paddle blades (C-, J-, O-, V-types) to move the tongue in different directions. The C-type blade is used for moving the tongue to the left and right. The J-type blade is used for moving the tongue to the posterior, while the V-type blade is used to move the tongue to the superior (i.e., palate). The O-type blade is used to move the tongue to the inferior, left, and right by changing the rotational angle of the paddle. Fig 2 shows the available positions for the paddle shaft in depth direction, the available tilt angles of paddle shaft, and rotational angles of the paddle blade. The device provides seven depth positions, three tilt angles, and eight rotational angles. In addition, a dedicated dry heat machine (BinkieHT^®^, Paprica Lab., LTd.) was used to heat the ethylene vinyl acetate (EVA) for the teeth impression [22]. Fig 3 shows the pCT images of the patients with a tongue-positioning device. The patients were placed in the supine position and scanned using a CT simulator (Brilliance Big Bore, Philips). Thermoplastic masks were used for immobilization. CT images with the commercial metal artifact reduction algorithm were acquired with a 3-mm-thick slice. The volume of interest, including the tongues, was contoured, and the volumetric modulated arc therapy plans were generated using the Eclipse version 16.1 treatment planning system (TPS) (Varian Medical Systems). AcurosXB version 13.7 were used. The grid size for dose calculation was set to 2 mm. RT was performed using a VitalBeam linear accelerator (Varian Medical Systems, CA, USA) with 6 MV X-rays. The margin from gross tumor volume to CTV was 5 mm, while the margin from CTV to planning target volume (PTV) was 3 mm.

**Table 1:** Patient characteristics and types of tongue positioning device.

Target	No. of patient	Prescription	Sex		Type of head of tongue device		Rotation angle of O-type (degree)	
**Nasopharynx**	6	67.5 Gy/30fx^a)^	Female Male	1 5	O-type	6	0	6
**Oropharynx**	21	67.5 Gy/30fx 63 Gy/28fx^b)^	Female	1	C-type	1	0	13
			Male	20	O-type	17	90	3
							270	1
					V-type	3	NA	NA
**Hypopharynx**	1	67.5 Gy/30fx	Male	1	O-type	1	0	1
**Nasal cavity**	2	67.5 Gy/30fx	Female	2	O-type	2	0	2
**Oral cavity**	3	67.5 Gy/30fx 63 Gy/28fx	Male	3	O-type	3	0	3
**Maxilla**	1	67.5 Gy/30fx	Female	1	O-type	1	0	1
**Parotid gland**	3	63 Gy/28fx	Female	2	O-type	3	90	1
			Male	1			270	2
**Salivary gland**	2	63 Gy/28fx	Female	1	O-type	1	90	1
			Male	1	V-type	1	NA	NA
**Neck node**	6	67.5 Gy/30fx	Female	2	C-type	1	0	2
		63 Gy/28fx	Male	4	O-type	5	270	3
**Tongue**	5	63 Gy/28fx	Female	3	O-type	5	0	5
			Male	2				
**Floor of mouth**	2	67.5 Gy/30fx 63 Gy/28fx	Female	1	O-type	1	0	1
			Male	1	V-type	1	NA	NA
**Total**	52		Female	14	C-type	2		
			Male	38	O-type	45		
					V-type	5		

a) The prescription for radical radiation therapy is 67.5 Gy with 30 fractions.

b) The prescription for adjuvant radiation therapy is 63 Gy with 28 fractions.

NA: Not applicable

### Tongue geometry

**Fig 4:**
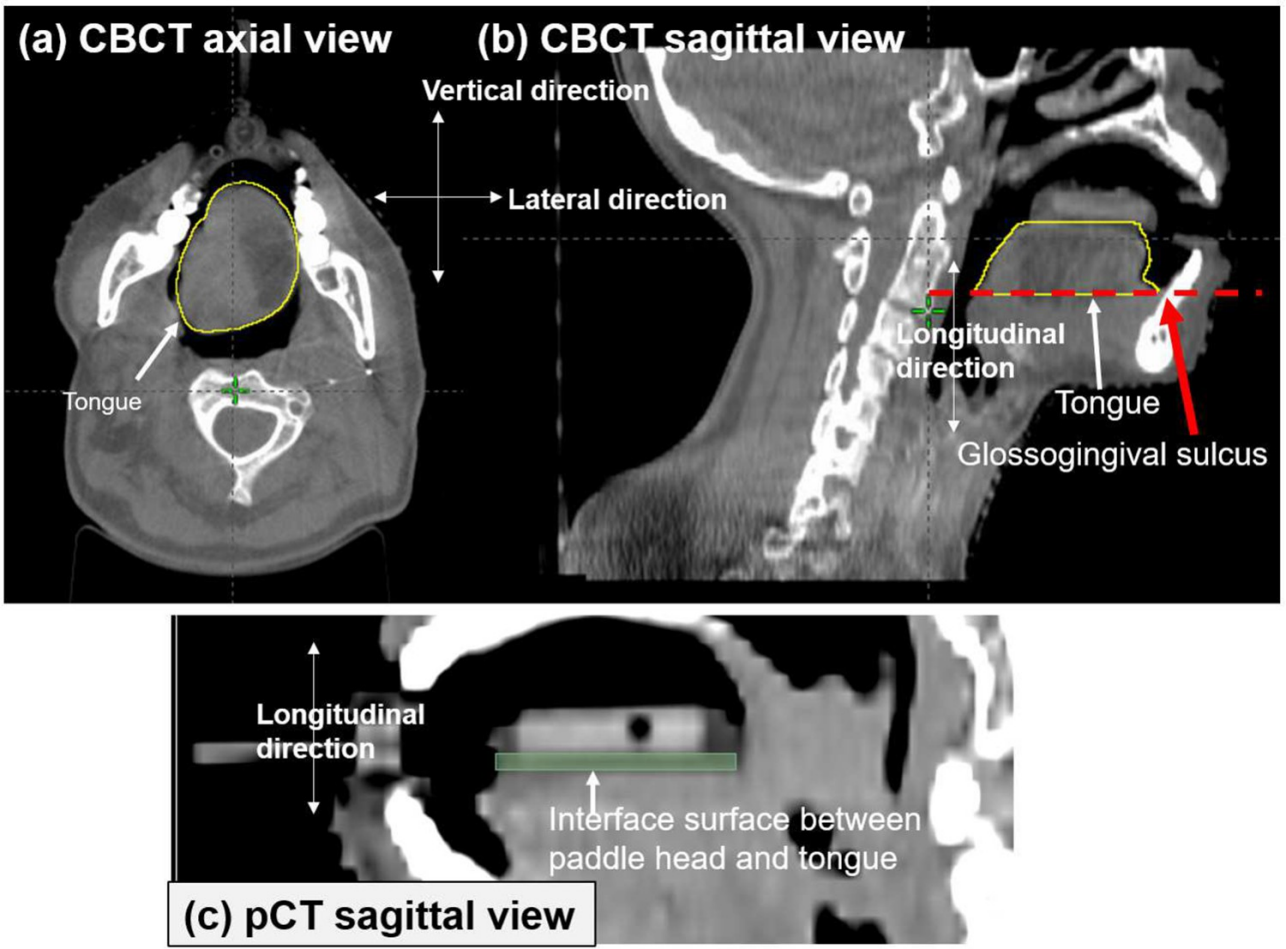
CBCT images of patients with tongue-positioning devices. (a) Axial view and (b) sagittal view of O-type tongue positioning device applied to a patient. The red dotted horizontal line shows the bottom plane of the tongue. (c) Example of contouring the surface between paddle blade and tongue for extracting the coordinates of radiation dose (RD).

The tongue is not a rigid structure. Therefore, it may be difficult to define the tongue structure based on a perfect consensus between radiation oncologists. Therefore, we defined the bottom plane of the tongue structure where the glossogingival sulcus is shown in the sagittal view [red arrows and red dotted horizontal line in Fig 3(C) and 3(D)]. CBCT scans were acquired at the 1^st^, 7^th^, 11^th^, 15^th^, 19^th^, 23^th^, and 27^th^ fractions. The CBCT images were rigidly registered to the pCT using online 3D/3D matching. After treatment, the tongue on the CBCT images in each patient was contoured in the TPS (Fig 4). The tongue structure in the pCT images in each patient was compared with that in the CBCT images at the 1^st^, 7^th^, 11^th^, 15^th^, 19^th^, 23^th^, and 27^th^ fractions. The geometrical differences in tongue volume between CBCT and pCT were analyzed using the Dice similarity coefficient (DSC) and average Hausdorff distance (AHD) using an open source software for visualization and analysis of medical image data sets, 3D slicer, which has been widely used for various applications in radiation oncology [23, 24]. DSC and AHD are widely used metrics for evaluating differences between two volumes. Many other studies provided detailed explanations of DSC and AHD [25, 26].

### *In vivo* dosimetry

*In vivo* measurements were performed once per week in 13 patients. To exclude the effect of imaging doses due to CBCT scanning, external beam therapy 3 (EBT3) films (Gafchromic; International Specialty Products, Wayne, NJ, USA) were attached to the head plate of the tongue device on the day when CBCT scanning was not performed [Fig 1(E)]. A waterproof tape was attached to the EBT3 films. The dimension of the EBT3 film was 2.0 × 2.5 cm^2^, while the dimension of the region of interest (ROI) was 1.6 × 1.6 cm^2^ because the pixels on the borders of the EBT3 films showed extremely high values due to the wet by the saliva. The EBT3 films were calibrated with 6 MV X-rays and analyzed in accordance with the TG-51 and TG-235 protocol [27, 28]. The value of the red channel from a scanned film image was used in the transmission mode and 300 dot-per-inch. The pixels of the measured dose distribution were produced to be 1.0 × 1.0 mm^2^. The calculated dose distribution in radiation dose (RD) file in the diagnostic imaging and communications in medicine format at the surface plane of the head plate of the tongue device was compared with the measured dose distribution. To extract the matched dose plane, the surfaces of the paddle blade interfacing with the tongue were contoured in the pCT images [Fig 3(C)]. The coordinates of the head-plate structure were used to extract the dose at the same location. The pixel size of the calculated dose distribution was also reduced to 1.0 × 1.0 mm^2^ by using a linear interpolation. The thickness of the RD plane was 1.5 mm.

**Fig 5:**
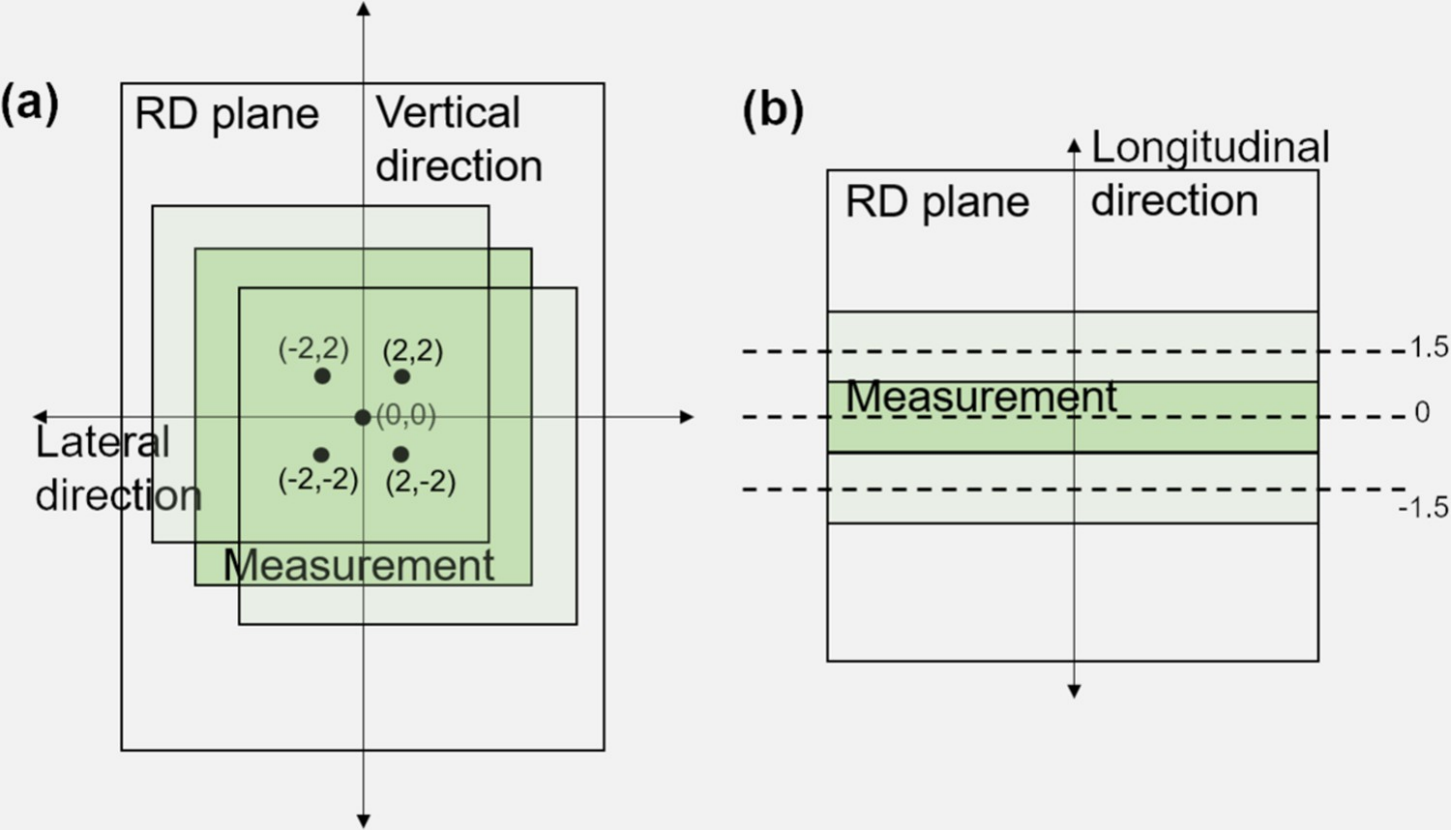
Shift of measured dose distribution. The measured dose were shifted in (a) the vertical and lateral directions and (b) longitudinal direction.

Without CBCT imaging, a patient setup was only conducted using wall lasers in the treatment room, which could result in setup errors. Such setup errors could hinder reliable dose comparison between the planned dose distribution and measured dose distribution. Therefore, we intentionally moved the measured dose distribution up to 2 mm in superior-interior (longitudinal), and in left-right (lateral) with 1 mm increment, and to 1.5 mm in anterior-posterior (vertical) directions with 1.5 mm increment (Fig 5). The range of this shift was determined based on the couch shift recording data of a four degrees of freedom couch. On-line 3D-3D matching was performed only in translational directions (i.e., vertical, lateral, and longitudinal directions), excluding rotation. The couch shift data were collected when CBCT images were acquired and matched with the pCT images (Table 2). A global gamma analysis with a 10% threshold was used to compare the 2D dosimetric differences between the measured and calculated dose distributions. The best gamma passing rate with 3%/3 mm criteria was saved among the 75 gamma analyses per pair of EBT3 film and RD considering the shifts (5 in longitudinal × 5 in lateral × 3 in vertical shifts) of the measured dose distribution.

**Table 2:** Average and standard deviation of couch shift in the vertical, lateral, and longitudinal directions. The couch shifts data were collected by on-line 3D-3D matching.

Couch shift	Vertical	Lateral	Longitudinal
**Average (mm)**	0.43	-0.29	-0.06
**Standard deviation (mm)**	1.22	1.44	1.11

### Statistical analysis

A paired t-test (*α* = 0.05) was used for statistical analysis. The DSC and AHD of tongue structures on pCT and CBCT at the 1^st^ fraction were compared with those of DSCs and AHDs between pCT and CBCT at other fractions to assess whether there were statistically significant differences. In addition, the gamma passing rate between the measured and calculated doses in the first week was compared with that in other weeks.

## Results

### Geometrical evaluation

**Fig 6:**
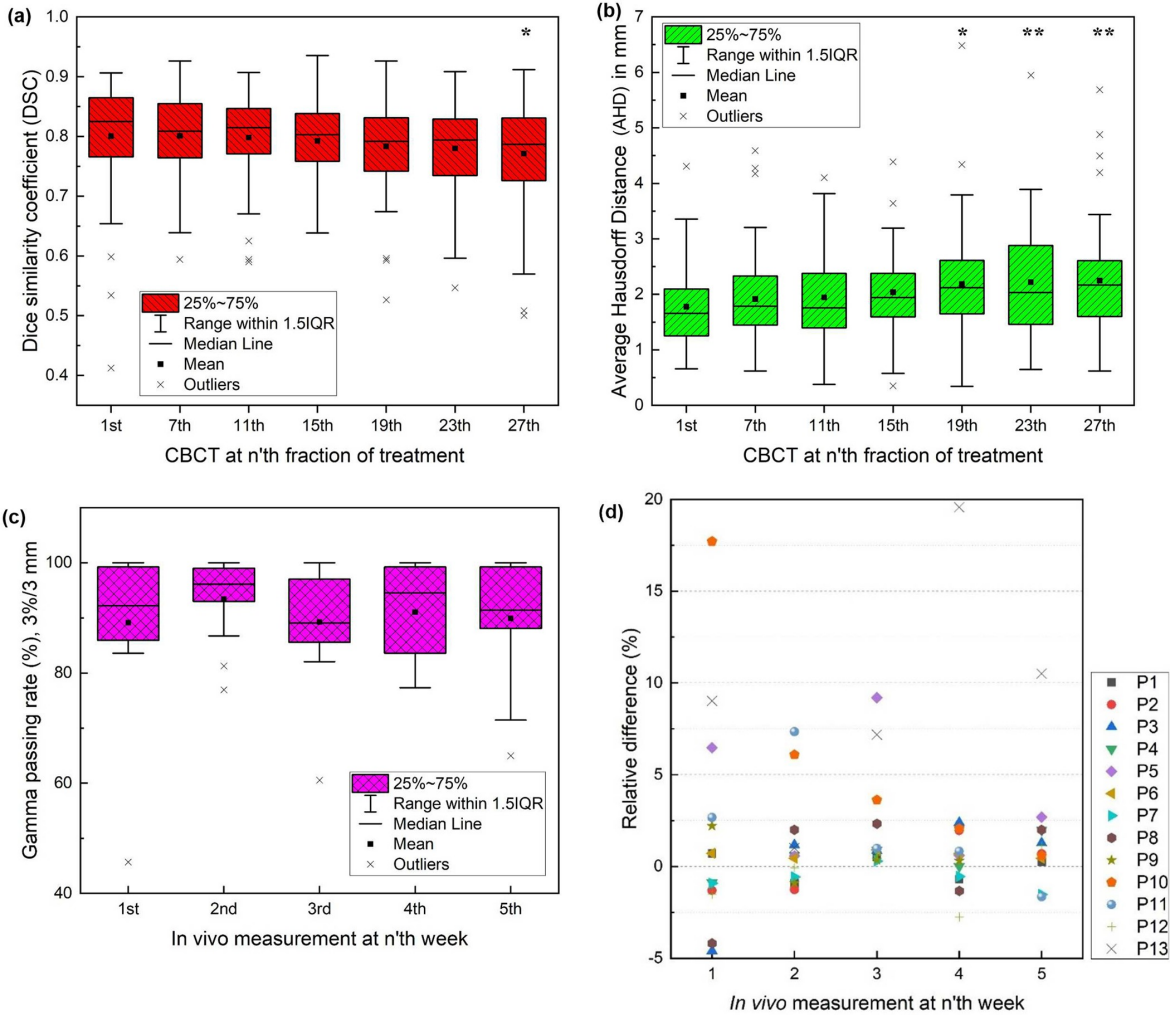
DSC and AHD between pCT and CBCT: Gamma passing rate and dose difference between the measured dose and calculated dose. (a) DSC and (b) AHD between pCT and CBCT at n’th fraction of the treatment. 25%~75% indicate the data between the first quartile (Q1) and the third quartile (Q3). (c) Gamma passing rate with 3%/3 mm criteria for in vivo external beam therapy 3 (EBT3) measurement at n’th week. 25%~75% indicate the data between the first quartile (Q1) and the third quartile (Q3). (d) The relative difference between the average measured dose and the average calculated dose with respect to n’th week of measurements. IQR indicate inter-quartile range, and IQR = Q3-Q1. Lower bound of 1.5IQR is Q1-1.5IQR and the upper bound of 1.5IQR is Q3+1.5IQR. The data less than the lower bound or more than the upper bound is outliers. * indicates a statistical significance of p-value < 0.05 and ** indicates a statistical significance p-value < 0.01.

Fig 6(A) and 6(B) shows the DSC and AVD between pCT and CBCT at n’th fraction of the treatment. Regarding DSC, the minimum was 0.77 at the 27^th^ fraction. The difference between the DSC of pCT and CBCT at the 1^st^ fraction and that at the 27^th^ fraction was statistically significant, with a *p*-value of 0.015. The relative difference between the DSC of the pCT and CBCT at the 1^st^ fraction and that at the 27^th^ fraction was -3.7%. Regarding AHD, the maximum was 2.25 mm at the 27^th^ fraction. The difference between the AHD of the pCT and CBCT at the 1^st^ fraction and these at the 19^th^, 23^rd^, and 27^th^ fractions was statistically significant (*p*-values: 0.019, 0.006, and 0.002, respectively). The relative difference between the AHD of the pCT and CBCT at the 1^st^ fraction and that at the 27^th^ fraction was greater than 26%. However, the absolute difference was within 0.5 mm in all fractions.

### *In vivo* dosimetry

**Fig 7:**
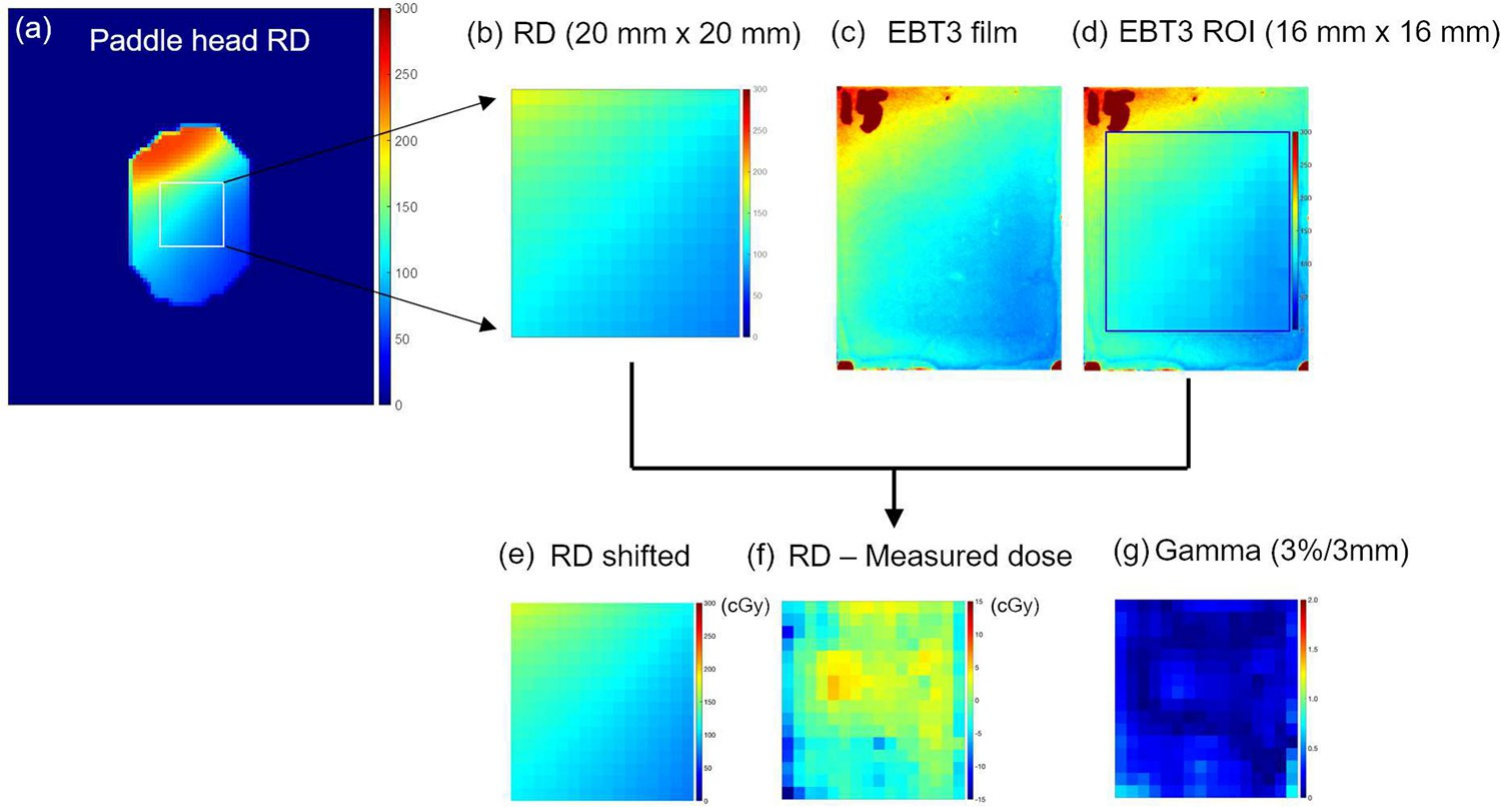
The measured dose and calculated dose distributions from a patient case. (a) The planned dose distribution (RD) on the paddle blade of the tongue positioning device. (b) RD in ROI 20 × 20 mm^2^). (c) The measured dose from external beam therapy 3 (EBT3) film on the paddle blade and (d) the measured dose in ROI (16 × 16 mm^2^). (e) Shifted RD which gamma passing rate with 3%/3 mm shows the highest value. (f) The dose difference between the shifted RD and the measured dose. (g) 2D Gamma between the shifted RD and the measured dose.

The intentional shifts of the measured dose distribution were saved and evaluated when the gamma analysis between the measured dose and the calculated dose yielded the best gamma passing rate (Table 3). Fig 6(C) shows the gamma passing rates with respect to the weeks of *in vivo* measurements. There was no significant difference (*p*-value > 0.05) between the gamma passing rate of the measured dose at the 1^st^ week and the calculated dose (RD) and the gamma passing rate of the measured dose at the other weeks and RD. The mean gamma passing rate in 13 patients ranged between 89.1% and 93.4% from the 1^st^ week to the 5^th^ week. The dose values on 2D films and the calculated doses (RDs) were averaged, and the difference between the average doses was compared. Fig 6(D) shows the relative dose differences between the average RD and the measured dose for 5 weeks’ measurements in 13 patients. Relative differences were mostly within ±3%. Some large discrepancies between the calculated and measured doses might be mainly due to patient setup errors. In addition, the doses in such cases were between 30 cGy and 40 cGy. Therefore, the relative difference could be large, even though the absolute difference was within a few cGy. Fig 7 shows the measured and calculated dose distributions in patient 2 (P2) who underwent RT for oropharyngeal cancer.

**Table 3:** Average and standard deviation of intentional shifts of measured dose distribution in the vertical, lateral, and longitudinal directions. The intentional shifts were saved and evaluated when the gamma analysis between the measured dose distribution and the calculated dose showed the best gamma passing rate.

Couch shift	Vertical	Lateral	Longitudinal
**Average (mm)**	-0.14	0.02	-0.54
**Standard deviation (mm)**	1.65	1.61	1.34

## Discussion

In this study, the geometrical changes in interfractional tongue structures were assessed by comparing the DSC, AHD, and *in vivo* measurements at several fractions between the 1^st^ fraction and 27^th^ fraction using a commercial tongue positioning device. As illustrated in Figs 1 and 2, the tongue positioning device has four types of paddle blades that shift the tongue to a specific direction. An O-type paddle blade can be used to press or shift the tongue to the left and right. Of note, the V-type paddle blade could raise the tongue and was useful when the PTV was the floor of mouth as described in Fig 3(D). The average DSC of pCT and CBCT at 1^st^ fraction and that at the 27^th^ fraction for five patients with V-type paddle blades were 0.82 and 0.80, respectively. The average of AHD of pCT and CBCT at 1^st^ fraction and that at the 27^th^ fraction for five patients with V-type paddle blades were 1.88 mm and 2.21 mm, respectively. The DSCs of pCT and CBCT at 1^st^ fraction for two patients with C-type paddle blades were 0.84 and 0.88, respectively. The DSCs of pCT and CBCT at 27^th^ fraction for two patients with C-type paddle blades were 0.74 and 0.79, respectively. The AHDs of pCT and CBCT at 1^st^ fraction for two patients with C-type paddle blades were 1.03 mm and 1.84 mm, respectively. The AHDs of pCT and CBCT at 27^th^ fraction for two patients with the C-type paddle blades were 2.11 mm and 2.81 mm, respectively. Due to the limited number of patients with C-type paddle blades, the statistical significance of the differences of DSCs and AHDs was not evaluated.

The limitation of the tongue positioning device is that it is not available for patient without teeth, because immobilization of tongue positioning device can be performed by impressing the teeth in the EVA part. The tongue is not a rigid structure, and it is easy to change its shape and volume periodically. We defined the bottom of the tongue using the glossogingival sulcus in the sagittal view, as described in Fig 3(C) and 3(D). The value of DSC itself can be affected by the definition of the bottom plane of the tongue structure, as the DSC is computed using volume information. If an unchangeable volume of tongue structure below the glossogingival sulcus is included, DSC can be overestimated. Therefore, the definition of the bottom plane of the tongue structure used in this study might be conservative and reasonable because we only considered the changeable part of the tongue structure. The DSC difference between 1^st^ and 27^th^ fraction was statistically significant; however its relative difference was only -3.7%. Since the average DSC at 27^th^ fraction was 0.77, we can conclude that the tongue was well immobilized and maintained a reproducible position even towards the end of treatments. AHD increased as treatment was continued. However, its distance was controlled within 2.25 mm compared with that in the CT simulation, and it was within the PTV margin (3 mm). When image-guided RT is not applied to the treatment of tongue cancer, the PTV margin can be determined by considering both the expected AHD and setup errors. DSC and AHD are useful metrics for assessing the volume coincidence between pCT and CBCT. However, they have limitations. pCT and CBCT have different spatial resolutions, and this difference may induce inherent differences when calculating the DSC and AHD. In this study, the voxel resolution of pCT was 1.37 × 1.37 × 3 mm^3^, whereas the voxel resolution of CBCT was 0.51 × 0.51 × 2 mm^3^.

It has been reported that dental prostheses can generate the scattered electrons, leading to an increase in dose of up to 170% compared to the dose without prostheses [29]. The use of tongue positioning device has been shown to reduce the scattered electrons originating from dental prostheses [30–32]. The mass density of EVA material used for teeth impression and paddle blades of BinkieRT^®^ is 0.96 g/cm^3^ and it has a similar Houndsfield unit (HU) to that of soft tissue. In pCT images, the HU range of the EVA material in the paddle blade was between -70 and 140, while the HU range of the tongue was between -20 and 70. Additionally, Baek et al. and Yoshizawa et al. reported that the customized 3D bolus in the oral cavity resulted in a better dose build-up effect [33, 34]. Huang et al. found that a 3D printed silicone bite block could reduce the dose to the adjacent normal tissues and improve dosimetric parameters such as dose homogeneity index and conformity index [35]. The BinkieRT^®^ device with the paddle head has potential to serve as a bolus for dose build-up and reduce the dose to the normal tissues. The BinkieRT^®^ has undergone and passed toxicity tests, including skin sensitization, acute oral mucosa irritation, and in vitro cytotoxicity tests following ISO 10993–5: 2009(E) and ISO 10993–10: 2010(E) standards.

In this study, we suggested the intentional shifts of the measured dose distribution in three directions to account for patient setup errors, aiming to achieve the best gamma passing rate. However, unlike 3D-3D matching, which focuses on the spatial difference, gamma analysis considers both dose and spatial differences. We assumed that the patient setup errors would decrease after implementing the intentional shifts of the measured dose. Since the gamma evaluation employed a 3 mm/3% criteria, an additional 3 mm in 2D distance to agreement (DTA) between the measured dose and the calculated dose was considered. The 3 mm DTA encompassed spatial differences arising from the displacement of tongue-positioning device, tongue motions and the patient setup errors which could not fully considered by intentional shifts alone. Some of the measured doses exhibited significant discrepancies compared to the calculated dose [Fig 6(D)], which could be attributed to patient setup errors. Despite applying measured dose shifts of ±2 mm in the vertical and lateral directions and ±1.5 mm in the longitudinal direction to accommodate setup uncertainties, there were instances where the setup error exceeded these shift ranges at certain fractions.

As reported in previous studies, the EBT3 film is known to have dosimetric uncertainties of 3.2% [19]. The sources of the uncertainties are the uniformity of the film, generation of the calibration curve, and type A measurement uncertainty. Reflecting the uncertainty of the reference beam calibration (0.9%) from the publication [36] and daily output uncertainty in our linear accelerator machine performance check (0.5%), the total uncertainty can be 3.4% at least. In addition to the uncertainty due to the EBT3 film, the dose calculation in TPS can be another source of uncertainty. The evaluated dose plane was located in the oral cavity region where the heterogeneity can affect the dose calculation accuracy. Nevertheless, the gamma passing rates with 3%/3 mm between the measured dose with shifts and calculated dose distributions were 89.1% to 93.4% in our study. The *in vivo* measurements in our study demonstrated that the dose at each fraction of measurement was delivered appropriately to the immobilized tongue with the tongue positioning device.

## Conclusions

Geometrical changes in the tongue during RT with a tongue positioning device for head and neck cancer were evaluated using pCT and CBCT. *In vivo* measurements using EBT3 films showed no significant interfractional differences between fractions. In conclusion, the tongue positioning device used in this study was found to minimize interfractional variations in the position and shape of the tongue.
